# Novelty, Consistency, Transparency: The Trilemma of Psychological Sciences and its Consequences on Open Science Practices

**DOI:** 10.5334/irsp.979

**Published:** 2025-03-21

**Authors:** Paul Bertin, Kenzo Nera

**Affiliations:** 1 Université libre de Bruxelles, BE

**Keywords:** meta-psychology, open science, novelty, consistency, transparency, virtue signalling

## Abstract

The past decade has emphasised the importance of transparency for robust psychological research. However, transparent research has a cost, and it is hardly compatible with both conceptual novelty and statistical consistency across multiple studies. We propose that these three criteria can be conceptualized as a trilemma: fulfilling two of them considerably reduces the probability of satisfying the third one. An article testing a novel idea and transparently reporting evidence is likely to include empirical failure that impede consistency. An article transparently reporting consistent findings probably will acknowledge a replication effort that does not seek theoretical advances. Finally, an article presenting consistent evidence through multiple studies for a novel idea is not likely to be transparent.

At a practical level, we argue that the pressure of the trilemma poses a threat for transparency, which is less tangible and historically important in the evaluation of research articles than the two other criteria. While the open science movement grows in importance, the pressure of the trilemma may encourage an opportunistic use of open science practices as a form of virtue signalling compensating for low transparency. Stakeholders, from editors to reviewers, should be aware of the constraints posed by transparency to continue improving the robustness of psychological science and avoiding a deleterious use of open science practices. We review potential solutions to break the pressure of the trilemma.

Psychologists face the growing expectation to be transparent to improve the robustness of their findings. While necessary, transparency has a double cost for researchers. At the practical level, it represents a supplementary workload (e.g., the implementation of open science practices; Hostler 2023). At the evaluative level, transparency is superimposed on existing criteria that remain crucial in the editorial assessment of articles. Indeed, publishing stakeholders, from publishers to reviewers, expect authors to report *novel* ideas (Tiokhin & Derex 2019; Tiokhin et al. 2021a) supported by *consistent* findings (Bakker et al. 2012; O’Boyle et al. 2017). Therefore, while requiring extra efforts, transparent research faces a publishing disadvantage when competing with non-transparent research (Vazire 2019). We argue that this situation is not only unfair but also epistemologically unrealistic and potentially deleterious for open science practices.

We propose that the compliance of empirical research with these three criteria – transparency, novelty, consistency – can be conceptualized as a *trilemma*. Fulfilling two of these criteria considerably reduces the probability of satisfying the third one. We propose that transparency is the only necessary criterion. When transparency is combined with either novelty or consistency, these pairs should often be sufficient to evaluate the scientific quality of an article, be it an original contribution or a replication. Furthermore, we argue that in a publishing system that would not compromise on either novelty or consistency, transparency is likely to be sacrificed.

In the following section, we define each term of the trilemma and review the evidence for its combinations. Then, we discuss how the pressure for novel and consistent results may lead to an opportunistic use of open science practices to compensate for low transparency. Finally, we review potential solutions to break the pressure of the trilemma.

## The terms of the trilemma

Jointly satisfying novelty, consistency, and transparency is hardly reachable within a single multiple-study article.^1^ Investigating newness, Shibayama and Wang (2020, p. 410) have defined originality as the ‘degree to which a scientific discovery provides subsequent studies with unique knowledge that is not available from previous studies’. At an empirical level, novelty is usually measured either by evaluating the uniqueness of knowledge elements, or the unusual recombination of already pre-existing knowledge elements (Shibayama et al. 2021). Novelty has also been studied by tracking changes of citations patterns to measure the impact of novel articles within a field (e.g., Park et al. 2023), but this level of analysis is out of the scope of our contribution, which focuses on novelty at the conceptual level. We propose that a novel idea whose empirical examination is transparently reported is likely to include some results that will not match the predictions, thereby hindering consistency.

Accordingly, we define consistency as the degree of correspondence between one’s reported predictions and research outcomes. In social psychology, authors typically predict a statistically significant difference in a certain direction without specifying effect size. However, predictions can also be based on more precise parameters, such as confidence intervals around an expected (or absent) effect (e.g., equivalence testing; Lakens et al. 2018). We primarily consider the former, more common type of directional predictions, but our argument similarly applies to more complex hypotheses.

By extension, we refer to consistency to designate the repeated validation of one’s hypotheses in an article reporting multiple studies – or multiple tests in the context of a single-study article. A contribution transparently reporting consistent findings is likely to be low on novelty; for example, it will test non-risky predictions or consist in replications.

Transparency in research is rarely defined and is often employed as self-explanatory or interchangeably with open science (e.g., The Second French National Plan for Open Science; MESRI 2021). Nevertheless, transparency is a complex concept, and some researchers have even proposed to use a taxonomy-based definition to cover the multi-faceted aspects of this term (e.g., Elliott 2022). An overarching definition of transparency could be ‘the principles and practices of being open about how a research study is conducted, with a full presentation of its assumptions, scope, processes, data, results, analyses, and implications’ (Hevner et al. 2024, p. 1).

**Figure 1:**
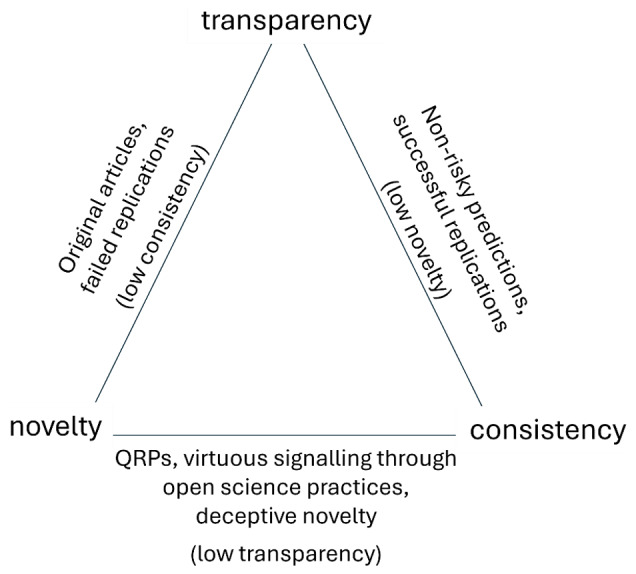
The Trilemma and its Outcomes. *Note*. QRPs = questionable research practices. We use the term ‘failed’ replication throughout the article for the sake of clarity but acknowledge that it may not be optimal, as it suggests that the problem lies in the replication rather than in the original study (LeBel et al. 2019).

Following the trilemma hypothesis, a contribution presenting novel *and* consistent findings is probably not transparent. One should note that a lack of transparency not only can manifest at the empirical level (e.g., file drawing failed studies and their preregistration forms to achieve an impression of consistency) but also at the conceptual level (e.g., deceptively presenting old ideas as new to achieve an impression of novelty). The trilemma is summarised in Figure 1, and evidence for each combination of the trilemma is reviewed in the next sections.

## The combinations of the trilemma

### Consistent novelty is the gold standard, but it is opaque

It is tempting for scholars to focus on novelty in developing their research agenda. Indeed, promoting new ideas by means of original articles is the norm for publishing in high-ranked journals in psychological sciences. Overall, novelty leads to increased article acceptance, generates more citations in the long run, and is key for obtaining funds and advancing careers (Teplitskiy et al. 2022; Wang et al. 2017). However, while valuing risky predictions, journals seldom tolerate null or even mixed findings and rather expect perfect-looking results (Giner-Sorolla 2012). In other words, journals tend to expect immaculate results through a spotless consistency of validated hypotheses.

The replication crisis that affected several psychological disciplines over the past decade reinforced and democratised the need to replicate studies to attest to the robustness of findings (Maxwell et al. 2015). Hence, a growing expectation has emerged for authors to self-replicate their findings through multiple studies to increase the weight of evidence for their ideas (Schimmack 2012). This expectation is well-illustrated by the fact that the average number of studies reported per article in some top social psychology journals has been increasing continuously (Sassenberg & Ditrich 2019). This trend was documented many years before the replication crisis (Wegner 1992), but the inflation of the number of studies per article has continued in recent years (Anderson et al. 2019). Novelty alone is no longer rewarding in contemporary psychological sciences; *consistent novelty* across studies is required to publish in prestigious outlets.

However, we view consistent novelty as hardly achievable if researchers are strictly transparent at the empirical and conceptual levels. At the empirical level, an article testing a new idea and reporting findings that consistently match predictions across studies is likely *not* transparent. Indeed, the conventional 80% statistical power in psychological research leaves room for Type 2 errors (i.e., false negative), especially in the case of multiple-study articles (Lakens & Etz 2017). For example, if three studies are conducted, given a conventional 80% statistical power, 5% Type 1 error rate, and assuming true effects, then the most probable outcome would be mixed findings (i.e., two out of three significant studies; see Lakens & Etz 2017). Thus, even if an effect exists in the population, it is unlikely to be detected consistently across multiples studies, especially given the sample and effect sizes that are typical in psychological sciences (Lovakov & Agadullina 2021). Making things worst, statistical power is often overestimated by psychologists (Bakker et al. 2016) and falls below 80% (e.g., 44% median power to detect medium effect sizes according to Szucs & Ioannidis 2017).^2^ Therefore, mixed findings are far from being as common as they should be in psychological sciences.

Often, empirical transparency is sacrificed through QRPs (Schimmack 2012) to maintain an illusion of statistical consistency between studies. Indeed, psychologists are surprisingly often right and successful in testing their published predictions. Scheel et al. (2021) demonstrated that while 96% of regular articles find support for their first tested hypotheses, this rate falls to 44% in registered reports, which are preregistered and accepted for publication before results are known. This difference in the rate of validated hypotheses is likely due to researchers being less transparent in traditional articles compared to the registered report format, which limits researchers’ degrees of freedom (Simmons et al. 2016). Similarly, Franco et al. (2016), compared discrepancies between published research and the related data made openly available by the funding programme that granted that research. They found that the median published *p* value was .02, while the median non-published *p* value was .35.

The chrysalis effect described by O’Boyle et al. (2017) refers to the variations in reported findings when dissertations are adapted to research articles. These changes, which include cherry picking among hypotheses and even the alteration of data, seek to improve the impression of consistency of the findings to increase the publishing value of articles. Such a quest for consistency has led authors to overly rely on heuristics for quantitative aspects of their research. This tendency to prove compliance with arbitrary thresholds is well illustrated by Cronbach’s alpha distribution being skewed over .70 – a practice labelled as ‘α-hacking’ (Hussey et al. 2025). The impression of consistency of novel empirical findings can also be altered through spin (i.e., ‘specific reporting that fails to faithfully reflect the nature and range of findings and that could affect the impression that the results produce in readers’; Boutron 2020, p. 432). This QRP has been found in 44% of abstracts in the biomedical literature (Boutron 2020) and 56% of articles in clinical psychology (Jellison et al. 2020).

Another way through which novelty and consistency can be achieved is at the expense of *conceptual* transparency. For example, such a deceptive impression of novelty can be achieved through strategic ambiguity (Frankenhuis et al. 2023) or by inflating the importance of some variables investigated while neglecting others (i.e., effect size magnification; Gandhi et al. 2024). Chhin et al. (2018) found that almost 49% of studies funded by the US Institute of Education Sciences could be considered conceptual replications, although none of them were presented as such. These findings speak against the anecdotal nature of deceptive novelty, which remains a key factor affecting the credibility of research (Hoekstra & Vazire 2021).

The contradictory injunction to provide an ever-increasing number of studies per article (Sassenberg & Ditrich 2019), while not adjusting the expectations in terms of validated hypotheses (Chopra et al. 2022), ultimately creates an incentive *against* transparency. By contrast, we argue that transparency should be the only necessary criterion – complemented by either novelty or consistency – to evaluate the quality of a contribution. Therefore, the only two realistic combinations of criteria imply that, for most contributions, either consistency or novelty will not be satisfied.

### Transparent consistency hinders novelty

Findings are more likely to be consistent when predictions are non-risky, that is, when hypotheses are relatively trivial or have a low degree of falsifiability (Lakens 2022a). Another case in which this high-consistency, low-novelty pattern is particularly likely to occur is in direct replication studies^3^ (or ‘exact’ replications; Zwaan et al. 2018). The goal of direct replication studies is to test the consistency of previously documented effects (LeBel et al. 2019). In essence, this type of contribution provides little novelty, making it adapted to the constraints posed by the trilemma hypothesis, assuming that transparency is met.

However, despite being a pillar of cumulative science (Clarke et al. 2024; Derksen et al. 2024; Simons 2014), direct replication studies are often disregarded due to their lack of novelty (Romero 2017). The consequence of such unpopularity is tangible, as replication articles remain rare in psychological science. For example, only 5% out of 250 sampled articles published between 2014 and 2017 reported replications (Hardwicke et al. 2022). Even more dramatic are the recent findings reported by Clarke et al. (2024): Only 0.2% of the total number of articles published in the top 100 psychology journal between 2010 and 2021 were replication studies. What is more, even when they are published, failed direct replications seem to have only a limited corrective effect on the literature, whether in terms of affecting citations of the original studies that failed to replicate (Schafmeister 2021) or the citation of the replication itself in posterior studies (Hardwicke et al. 2021). However, these findings have recently been tempered by a long-term analysis of the impact of failed replications on the citation pattern of original papers, which suffered an average citation decline of 14% (Clark et al. 2023).

A common way to maintain novelty while preserving an impression of consistency is through *conceptual* replications and replication *and extensions*, which are often reported within articles to attest to the robustness of findings (Kunert 2016). Going beyond resampling, these forms of replication seek to restrict the generalisability of an effect (Lakens 2022b), which is achieved by varying components of experiments, whether it is the operationalisation of a variable or the variable itself (Hudson 2023). However, these non-direct forms of replication do not offer the same level of evidence as direct replications do regarding the robustness of the original effect (Kunert 2016). Since conceptual replications are aimed at bringing some conceptual or methodological novelty (see Vermeulen et al. 2024), consistency is under greater pressure than in the context of direct replications. Furthermore, conceptual replications are frequently internal replications and, therefore, are conducted by the same team of researchers – a factor known as increasing the risk of conflict of interest and biased findings (Romero 2020).

For these reasons, conceptual replications are more at risk of QRPs than direct replications, just as any original study seeking conceptual or methodological advances. The similarity and blurred boundaries between conceptual replications and original studies is well captured in the fact that authors sometimes present conceptual replications as original contributions (Chhin et al. 2018). Furthermore, Kunert (2016) compared the replication rate of the effects tested in the Open Science Collaboration (2015) with internal replications of these effects in the original articles, most of which were conceptual replications. The success (or failure) of internal replications was not predictive of independent replications by the Open Science Collaboration (Kunert 2016). Therefore, it is crucial not to value conceptual replication over direct replication for the lure of novelty. Despite their lack of novelty, adequately constructed and powered direct replication studies offer a reliable form of consistency, which is key to ensure the robustness of psychological science’s empirical foundations (Zwaan et al. 2018).

Interestingly, direct replication studies present no novelty *if the hypotheses are validated*. Indeed, if the hypotheses are not corroborated, then a novelty feature will emerge from the deviation with the original findings (see, for instance, this failed replication of classic behavioural priming studies; Doyen et al. 2012). Consequently, contrasting with successful direct replications achieving transparency and consistency (but not novelty), contributions reporting failed direct replication meet transparency and novelty (but not consistency). This latter pattern of the trilemma is similar for original articles that transparently report mixed findings.

### Transparent novelty hinders consistency

Consistency is often set as a primary expectation by authors, editors, or reviewers when developing or evaluating an empirical article that presents a new idea tested through multiple studies (Franco et al. 2014; Muradchanian et al. 2023). However, this tendency is epistemically dubious, since it is natural for new ideas to evolve, for concepts to be refined, and for empirical examinations to face failures and report null results. This aversion for null findings has led to the notorious file drawer problem (Rosenthal 1979) which describes the tendency to only publish successful studies and hide the failed ones in the file drawer. This raises the alarming possibility that ‘journals are filled with the 5% of the studies that show Type I errors, while the file drawers back at the lab are filled with the 95% of the studies that show nonsignificant (e.g., p > .05) results’ (Rosenthal, 1979, p. 638). Rather than expecting immaculate evidence, the focus should be shifted to methodological rigour and valuing the *transparent novelty* of a contribution, which is how one’s novel ideas are presented as the fruit of an evolving intellectual process, supported by a set of almost necessarily mixed findings (Lakens & Etz 2017).

Of course, relaxing constraints on the consistency of findings does not amount to a blind acceptance of all studies. The informative value of a poorly designed, underpowered, and psychometrically poor study will remain low, regardless of its results. Similarly, one may question the scientific value of a methodologically sound study that is not grounded in any theoretical framework or even grounded in a blatantly false theory. For example, a recent meta-analysis demonstrated that a null field such as homoeopathy studies can reflect positive meta-analytic effects while relying on no theoretical foundation, thus reflecting the magnitude of bias in science (Sigurdson et al. 2023). The scientific value of studies must be considered regardless of their outcomes as long as they are theoretically sound and satisfy methodological standards (Devezer et al. 2021; Schimmack 2012). Research must not be valued based on its outcomes but on its methods and underlying theory. If results conflicting with researchers’ expectations negatively affect the evaluation of their work by their peers, the trilemma might lead to detrimental consequences.

## The consequences of the trilemma

Against the trilemma hypothesis, one could argue that it is ultimately possible to conceive an article that transparently reports findings that are both novel and that consistently validate one’s hypotheses. In fact, one needs only luck – for instance, by discovering an exceptionally large and non-trivial effect or by avoiding random sampling variations that produce false negatives when conducting multiple studies. To this objection, we answer that although such a novel and consistent set of transparent findings is technically possible, it is not desirable as a norm for psychological science. Indeed, expecting all manuscripts to comply with these three criteria as a norm, without revising expectations in the light of the cost of transparency, may have detrimental consequences.

Not conceding on novelty or consistency, expectations may turn open science practices into a varnish aimed at opportunistically embellishing publications with a misleading appearance of transparent reporting. If authors are urged to present perfect-looking, novel results in a publishing environment in which the open science movement is growing in importance, they might only *strategically* integrate open science practices into their contributions. In other words, ostensible adhesion to common open science practices, such as preregistration and data sharing, can become a form of virtue signalling.

Indeed, while transparent research requires open science practices, open science practices do not guarantee transparency.^4^ Unlike consistency and novelty, many aspects of research transparency are not tangible. An editor evaluating a manuscript can directly assess whether a set of studies repeatedly reports significant effects or whether an idea presented is innovative, but they cannot directly assess transparency. They can rely only on indirect clues indicating transparency, such as the presence of inconsistent findings (e.g., null or reverse findings), or open science practices (e.g., public availability of data files, analyses scripts, preregistrations).

Satisfying transparency by reporting failures will rarely lead to evaluative benefits and might even contribute to some depreciating of an article (Vazire 2019). By contrast, signalling transparency through strategic open sciences practices – for example, selectively granting open access to the data of the studies that yielded the expected results but not the studies that were relegated to the file drawer – might lead to benefits such as increased citations (Colavizza et al. 2024) while not conceding on novelty and consistency. Open science badges may be central to such a strategic use of open science practice to gain editorial and academic benefits while compensating for a lack of transparency due to the trilemma pressure.

First introduced by *Psychological Science* in 2014 (Eich 2014) and supported by the Center for Open Science (COS) *–* which claims the participation of more than 100 journals in this programme (COS 2024) – badges are pictograms attached to articles to certify the use of open science practices (i.e., preregistration, open data, and open materials). Badges are said to be effective in increasing transparency, for example, by increasing data sharing (Lindsay 2019). The direct and explicit goal of open science badges is to *signal* open science practices to readers (COS 2013), and some have argued that badges favour science through virtue signalling and reputational rewards (Kraft-Todd & Rand 2021). This approach to open science and badges advocates incentives based on media attention (Zong et al. 2023), self-promotion, and personal benefits (McKiernan et al. 2016), such as gaining additional citations by appearing more replicable (Kraft-Todd & Rand 2021).

However, open science badges should not be viewed as absolute transparency testimony. For example, many preregistrations are too vague to properly restrict researchers’ degrees of freedom (Bakker et al. 2020), leaving room for undisclosed deviations (Claesen et al. 2021) and selective reporting (van den Akker et al. 2023). Ultimately, preregistrations are often of poor quality, making them barely useful (if at all), yet articles reporting poorly preregistered studies benefit from the advantages offered by the open science label. Similarly, the open data badge seems to be poorly predictive of the reproducibility of studies published in *Psychological Science* (Crüwell et al. 2023), and studies with a preregistered badge seem to suffer as much of from p-hacking and HARKing (hypothesising after the results are known) than non-preregistered studies (van den Akker et al. 2024). Finally, authors have raised concerns regarding the role of scientific publishers in shaping this opportunistic and profit-oriented vision of open science (Fillon et al. 2024) as well as the poor attention given to open science practices, such as preregistration, by reviewers and editors (Syed 2023). Therefore, open science practices may be treated as an empty gesture not only by authors but also by other stakeholders of the publishing system, who benefit from keeping articles conceptually attractive and statistically immaculate while appearing to endorse a progressive vision of scientific research.

Such a potential opportunistic use of open science practices may explain why the rates of validated hypotheses (Scheel et al. 2021) and positive p-values (Böschen 2023) remain surprisingly high, despite the growing adherence to open science psychological sciences (Hardwicke et al. 2024). This form of virtue signalling through open science practices is also well exemplified by recent alleged fraud cases, in which the fraudulent data had been made available on the Open Science Framework by the authors (e.g., The Francesca Gino case; Simonsohn et al. 2023).

## Breaking the pressure of the trilemma

Beyond keeping the cost of transparency in mind when considering an article for publication, how can the pressure of the trilemma be alleviated? Below, we review some existing tools that may limit the constraints previously described. We organised these potential solutions into two categories: advances through technical settings and solutions acting at a social level through normative appeals. The proposed categorization is summarised in Table 1.

**Table 1:** Some Solutions to Break the Pressure of the Trilemma.


TECHNICAL SOLUTIONS		NORMATIVE SETTINGS	
	
WILLINGLY ADOPTED	EXTERNAL CONSTRAINT	INCENTIVES	SOCIAL SUPPORT

Registered report	Prospective registration	Open science badges	Open science communities

Machine-readable analyses	Measures to improve Honest signalling	Scientific awards	Educative programmes

Result-free review		Replication grants	Citing replications


### Technical solutions

Technical solutions are formalized procedures surrounding the publication process that can help overcome the pressure of the trilemma. These solutions can be adopted willingly by researchers (e.g., because they believe that doing so would improve the quality of research) or be externally enforced by regulators.

#### Willingly adopted technical solutions

The registered report format is among the most widespread willingly adopted solutions to protect transparency from bias and thereby enhance the credibility of research. Since articles are accepted before the results are known, this method enables the researcher to preserve novelty and transparency, at the cost of consistency (Nosek & Lakens 2014; for a complete overview of this format, see Chambers & Tzavella 2022). Registered reports successfully diminish bias related to the pressure for consistency, such as selective reporting and publication bias (Lakens et al. 2024), while maintaining a level of novelty and creativity comparable to traditional articles (Soderberg et al. 2021). Registered reports are also more reproducible and are more aligned with other open science practices than traditional articles (Obels et al. 2020). However, researchers perceive this format as time-consuming (Briker & Gerpott 2024), a limitation that the second generation of registered reports seeks to address (Eder & Frings 2021).

Another willingly adopted technical solution is machine-readable analyses, which, according to Lakens and DeBruine (2021, p. 2), consist in making computers evaluate ‘whether a statistical prediction is corroborated (or not) on the basis of clearly articulated evaluation criteria and the observed data’. Such a method has the potential to increase the rigour and transparency of analyses by mitigating researchers’ bias in the examination of their hypotheses (Guest & Martin 2021).

Results-free reviews represent another way to mitigate the pressure of the trilemma. In results-free reviews, the results section is written but is hidden from the editors and reviewers (Button et al. 2016). To date, this innovative form of reviewing has received only scant attention, but a sample of editorial board members determined that the method could positively affect the behaviour of authors and reviewers (Woznyj et al. 2018).

#### External constraints

Switching to external constraints, one of the best examples comes from the regulation of clinical trials. Indeed, mandatory prospective registration has been proved to be effective in reducing publication bias in clinical trials in the United States (Kaplan & Irvin 2015), and such a requirement could have similar impacts in psychological sciences (Thibault et al. 2023). Relatedly, it is also worth noting that solutions proposed by Tiokhin et al. (2021b) to limit deceptive research based on honest signalling include the intervention of externally enforced constraints, such as limiting the number of possible submissions or making rejections costly (e.g., by setting a delay between rejection and resubmission).

### Normative settings

As is true of anyone, researchers’ behaviour is largely determined by social norms and peer influence (Latour & Woolgar 1986), and open science practices and transparency ultimately are a set of behaviours in which people may or may not engage (Robson et al. 2021). Therefore, other ways to break the pressure of the trilemma may rely on incentives and social support aimed at shifting the normative setting in which researchers evolve.

#### Incentives

Open science badges are incentives, because they reward commitment to open science practices by making such adherence visible and, consequently, a valuable asset for researchers. In turn, this increased visibility may influence peers to engage with open science practices (Kraft-Todd & Rand 2021). However, this tool suffers from several flaws which can be attributed to badges becoming a goal in and of themselves (Klonsky 2024). A less controversial incentive may be scientific awards that recognise open science and replication efforts (e.g., Organization for Human Brain Mapping 2024). Encouraging these initiatives is crucial, because transparency is currently poorly acknowledged by award assessment criteria (Lagisz et al. 2024). Moreover, funding of replications remains rare (Chhin et al. 2018).^5^ The effectiveness of such distinctions in efficiently increasing transparency – especially at the community level, beyond the award winner – has yet to be demonstrated, which constitutes an important venue for future research.

#### Social Support

Lastly, some solutions to break the pressure of the trilemma rely on the social support and education of researchers (Azevedo et al. 2022; Kohrs et al. 2023) and reviewers (Köhler et al. 2020). The development of such open science communities aims to promote open science practices among peers and policy makers through local grassroot networks, with the goal of ultimately making open science the default norm. The implementation of open science coordinators in universities may also be useful to facilitate transparent research at a local level. Additionally, researchers may participate in low-cost efforts to support peers who are engaging in transparency, for instance, by ensuring that replications are integrated in the literature review. Merely citing others’ replication efforts will make engagement in replication efforts more visible and, therefore, more rewarding (Koole & Lakens 2012).

In the end, transparency can hardly be perfectly ensured based on technical constraints and normative settings, because those can always be misused. Cheaters will always be cheaters.^6^ Ultimately, the best guarantee and cost-efficient solution for transparency remains the authors’ good will – which would be best achieved in a research environment that would disincentivize non-transparent research.^7^

## Conclusion

In this contribution, we have argued that the current gold-standard criteria for publishing in a prestigious outlet – in other words, the conceptual novelty and statistical consistency of findings – are hardly reachable at scale in a scientific system that values transparency. Hence, the publishing standards must be revised according to the cost of the only necessary (but not sufficient) criterion, transparency. It is epistemically sound for original articles testing novel ideas to report failed hypotheses impeding the impression of consistency and for replication studies with a high consistency value to lack novelty. Such a trade-off would protect open science practices from becoming a form of virtue signalling compensating for a sacrificed transparency. Therefore, it is crucial to break the pressure of the trilemma through realistic reviewing and editing, promoting technical constraints such as registered reports and normative settings such as open science communities.

## Footnotes

We focus on empirical articles reporting a set of quantitative studies conducted with a frequentist approach, which we see as the norm for publishing in high-ranked psychology journals.

Note that recent research documented an overall improvement of the statistical power of studies in eight top social and personality psychology journals (Fraley et al. 2022). Such an increase of statistical power could mitigate the apparent contradiction between the inflation in the number of studies, and statistical consistency. The recent improvement in the replicability index of *Journal of Experimental Social Psychology* illustrates this point well (Soto & Schimmack 2024).

Although an important lesson from the recent replication database is that replication effect sizes are on average smaller than original effect sizes (Röseler et al. 2024). Therefore, even though successful replications can be perceived as yielding consistency in terms of directionality, their effect sizes are often not consistent in terms of magnitude and precision (see LeBel et al. 2019).

In fact, assuming so would amount to committing the formal fallacy of affirming the consequent: concluding based on conditional statement ‘if transparency, then open science practices’ that ‘if open science practices, then transparency’.

It is worth mentioning the funding scheme of the Dutch funding agency, the Netherlands Organisation for Scientific Research, as a notable exception (Derksen et al. 2024).

We thank the reviewer for this eloquent summary of these discussions.

The anonymous reviewer should be credited for this insight as well.
